# Higher hemoglobin levels are an independent risk factor for gestational diabetes

**DOI:** 10.1038/s41598-022-05801-y

**Published:** 2022-01-31

**Authors:** Niina Sissala, Sanna Mustaniemi, Eero Kajantie, Marja Vääräsmäki, Peppi Koivunen

**Affiliations:** 1 Biocenter Oulu and Faculty of Biochemistry and Molecular Medicine, Oulu Center for Cell-Matrix Research, University of Oulu, P.O. Box 5400, 90014 Oulu, Finland; 2 PEDEGO Research Unit, MRC Oulu, Oulu University Hospital and University of Oulu, Oulu, Finland; 3 Population Health Unit, Finnish Institute for Health and Welfare, Helsinki and Oulu, Finland; 4 Department for Clinical and Molecular Medicine, Norwegian University of Science and Technology, Trondheim, Norway; 5 Children’s Hospital, Helsinki University Hospital and University of Helsinki, Helsinki, Finland

**Keywords:** Physiology, Endocrinology, Medical research, Risk factors

## Abstract

Incidence of gestational diabetes (GDM) has increased rapidly. It poses significant risks for both mother and fetus affecting also negatively their longer-term metabolic heath. We asked whether early pregnancy maternal hemoglobin (Hb) levels, indicative for tissue oxygenation, would affect mother’s metabolic health and fetal outcome. We assessed in FinnGeDi, a Finnish multicenter case–control study for GDM (n = 1828), association of maternal 1st trimester Hb levels with metabolic parameters and perinatal outcome. Our data show that mothers with GDM had higher Hb levels compared to controls (mean difference 1.746 g/L). Hb levels associated positively with pre-pregnancy body mass index (BMI), fasting glucose levels and glucose levels in a glucose tolerance test and systolic and diastolic blood pressure (bp) levels. When assessed in quartiles the highest Hb quartile had more chronic and gestational hypertension and the most adverse outcome of the metabolic parameters, dose-dependency seen in bp, BMI and glucose levels. In a multivariable regression analysis Hb levels remained an independently associated parameter for GDM after adjusting for key covariates (OR 1.019, 95% CI [1.007; 1.031]). In conclusion, higher maternal Hb levels within the normal variation are an independent risk factor for GDM in this population but have little effect on perinatal outcome.

## Introduction

Gestational diabetes mellitus (GDM) is an increasingly common public health and clinical problem complicating pregnancy^1,2^. The global prevalence varies by countries being 2–25%, the current prevalence in Finland being 20.6%^3–6^. GDM is characterized by hyperglycaemia that develops during pregnancy and resolves after birth^2^. The diagnosis is typically based on a pathological glucose tolerance test. The established risk factors include maternal overweight and obesity, higher maternal age, previous history of GDM, family history of type 2 diabetes mellitus (DM) and ethnicity^2^. GDM poses significant short-term and long-term adverse health outcomes for both mother and child reinforcing the importance of acknowledging the risk factors of GDM, in particular the modifiable factors, and preventing the condition^1,7,8^. The established maternal short-term risks associated with GDM include increased rates of gestational hypertension and pre-eclampsia, and an increased frequency of Caesarean sections^7^. Macrosomia is the most significant risk for the fetus associated with an increased risk of shoulder dystocia and perinatal hypoglycaemia^7^. In longer term, women with a history of GDM have an increased risk of DM, metabolic syndrome and cardiovascular diseases^1,7,8^. Increased risk for developing obesity and other metabolic disorders and vascular diseases have also been reported for the offspring of mothers with GDM in longer term^1,8^. Nutritional therapy and increased physical activity are the cornerstones of GDM care and are generally recommended as the primary treatment options^7^. Medical treatment of glucose levels should be started if normoglycaemia is not achieved with lifestyle changes^7^.

Hemoglobin (Hb) is the main carrier of oxygen. Hb levels are regulated genetically and environmentally and they differ by sex, race, age, altitude and smoking^9,10^. Individual Hb levels during adult life are however relatively stable. During pregnancy, the up to 50% increase in maternal plasma volume without an increase in the number of red blood cells results in physiological anemia. According to World Health Organization Hb levels < 110 g/L during pregnancy are considered anemic^11^. In general, high-end Hb levels within the normal range are considered beneficial for health^10^. However, higher Hb levels in non-pregnant adults have been associated with obesity, adverse metabolism, higher blood pressure (bp) levels and increased inflammation^12–14^, all associated with GDM, suggesting that indeed lower Hb levels within the Hb reference range may associate with healthier maternal metabolism during pregnancy. Previous studies with small selected Asian and Turkish populations have associated higher 1st trimester maternal Hb levels with an increased risk for GDM, pre-eclampsia and gestational hypertension^15–18^. The largest so far study with > 20,000 Chinese non-smoking singleton participants also associated higher early pregnancy Hb levels with increased risk for GDM and pre-eclampsia while no information about the newborn parameters were reported^19^.

The aims of this study were to evaluate the association of maternal Hb levels with altogether > 30 maternal and perinatal outcome parameters in a Finnish multicenter case–control study for GDM. We set out to evaluate whether 1st trimester maternal Hb levels are associated with the risk of GDM independent of key sociodemographic and pregnancy-related covariates. We also set to compare the association of Hb levels in quartiles with the metabolic and perinatal outcome among the whole population and the participants with GDM only. Increased information about the risks for GDM will eventually lead to better maternal and child health through improved primary prevention.

## Materials and methods

### Study population

The present study is based on the clinical-genetic arm of The Finnish Gestational Diabetes study (FinnGeDi), a multicenter case–control study (for details see^20,21^). FinnGeDi has a register-based and a clinical-genetic arm. Data to the clinical-genetic arm was collected from February 2009 to December 2012 in seven Finnish delivery hospitals. Altogether 1146 women with singleton pregnancies diagnosed with GDM were recruited to the study in delivery units before delivery, and the next consenting mother without GDM giving birth in the same hospital was recruited as a control (n = 1066) (Fig. S1). Comprehensive, detailed data on pregnancy and delivery were collected from the hospital and the maternity clinic records by trained research nurses and medical students. Data was supplemented by individually linked register data obtained from the Finnish Medical Birth Register (MBR) and self-filled questionnaire. All methods were performed in accordance with the relevant guidelines and regulations.

An oral glucose tolerance test (OGTT) is recommended for screening of GDM in every pregnant woman, except those in a very low-risk group based on the National Finnish Current Care guidelines introduced in 2008^22^. The low-risk groups include < 25-year-old primiparous women with BMI < 25 kg/m^2^ without family history of DM and < 40-year-old multiparous women with BMI < 25 kg/m^2^ without history of GDM or a macrosomic newborn (birth weight > 4500 g). OGTT was mainly performed between the 24th and 28th gestational week, except for those in high-risk groups (prior GDM, BMI ≥ 35 kg/m^2^, glucosuria, family history of DM or polycystic ovary syndrome) it was recommended to be performed between the 12th and 16th gestational week. In cases in which the first OGTT of a risk group mother was normal, it was repeated between the 24th and 28th gestational weeks. The diagnostic criteria for GDM was plasma glucose concentration ≥ 5.3 mmol/l after overnight fasting, ≥ 10.0 mmol/l at 1 h of OGTT, or ≥ 8.6 mmol/l at 2 h of OGTT. The diagnosis of GDM was based on one or more abnormal values in OGTT. Additionally, GDM diagnosis was based on glucose self-monitoring in case of 24 participants^21^.

Inclusion criteria for the present study was Hb level within Finnish reference values for women (117–155 g/L)^23^. When gestation is considered the lower limit decreases to 110 g/L, making the reference Hb ranged in the present study from 110 to 155 g/L. The 1st trimester Hb value was counted as an average of the values measured before gestational week 13 to minimize variation. Participants were excluded for (i) missing 1st trimester Hb values (n = 321) or (ii) having Hb levels of > 155 g/L (n = 33) or (iii) < 110 g/L (n = 30). Altogether 1828 participants met the inclusion criteria of whom 958 were diagnosed with GDM and 870 were non-GDM controls (Fig. S1). The study population was divided into four quartiles according to the 1st trimester Hb levels: Hb quartile 1 (110–126 g/L), Hb quartile 2 (126.33–132.50 g/L), Hb quartile 3 (133–138.50 g/L) and Hb quartile 4 (139–155 g/L) (Fig. S1). When Hb quartiles were formed including only participants with GDM the Hb levels were: Hb quartile 1 (110–128 g/L), Hb quartile 2 (128.50–134 g/L), Hb quartile 3 (134.50–140.50 g/L) and Hb quartile 4 (141–155 g/L).

Each participant gave a written informed consent to participate in the FinnGeDi study after thorough explanation of the purpose of the study. The Finnish Institute for Health and Welfare (THL) holds the database, and the access is granted through ethics approvals and individual consent and, for register data, by the registry authorities. The data has been acquired according to Finnish Act on Medical Research (488/1999 and 295/2004) and the study has been approved by Ethics Committee of Northern Ostrobothnia Hospital District in 2009.

### Measures

Hb levels were measured with a point-of-care-test during the visits to the maternity clinic and values were obtained from the mother’s maternity clinic record. Information about the OGTT results and pharmacologically (insulin or metformin) treated GDM were obtained from mother’s medical records. For systolic and diastolic bp the highest and the second highest values before and after 20th gestational week were collected from the mother’s medical records. Diagnoses of hypertensive disorders were based on the medical records. Hypertension was defined as systolic bp ≥ 140 mmHg and/or diastolic bp ≥ 90 mmHg repeatedly. Hypertension was considered as chronic if it appeared before 20 gestational weeks or if the participant used antihypertensive medication before mid-pregnancy. Pre-eclampsia was defined when hypertension appeared after 20 gestational weeks combined with proteinuria (≥ 0.3 g protein/24-h urine specimen or two ≥ 1 + readings on a dipstick test). Gestational hypertension was considered when hypertension appeared after 20 gestational weeks and no proteinuria occurred. Maternal educational attainment was categorized as basic or less, secondary, lower-level tertiary and upper-level tertiary and obtained from self-filled questionnaire. The educational data was missing from 212 participants.

Pre-pregnancy BMI was calculated from the participant’s pre-pregnancy height and weight (kg/m^2^), which were obtained from the maternity clinic records. Maternal age at the delivery, parity, gravidity and smoking during pregnancy were obtained from the MBR. Newborn parameters including gestational age at birth, birth weight, Apgar scores, umbilical artery and vein pH were obtained from the MBR. Placental weight, placental weight ratio, need for any supplementary oxygen or respiratory support (including everything from a short-term oxygen supplementation in delivery room to assisted respiration), need for glucose infusion, length of hospital stay were obtained from the child’s medical records. The birth weight standard deviation (SD) score is a sex- and parity-specific parameter estimating birth weight and length in singletons born at 23–43 gestational weeks, according to Finnish standards^24^.

### Statistical analysis

Categorical variables are reported as numbers and frequencies (%) and continuous variables as means with SD. To characterize the differences between GDM and non-GDM groups Student’s two tailed *t* test was used for continuous variables and chi-square test for categorical variables. Pearson’s correlation coefficient was calculated to compare linear dependencies between Hb levels and maternal metabolic and other parameters and perinatal outcome. To adjust for BMI partial correlation was analyzed. Approximate 95% confidence intervals for partial correlation were calculated using z-score transformation and linear regression.

To assess the differences between the four different Hb quartiles one-way ANOVA was used for continuous variables and chi-square tests for categorical variables. To adjust for potential confounding factors; smoking, the gestational week Hb was measured, pre-pregnancy BMI and chronic hypertension, a general linear model (GLM) was used. A *p* value ≤ 0.05 was considered statistically significant. *p* values < 0.001 were not given as exact values.

Linear regression (mean difference with 95% confidence interval (CI)) was used for estimating the effect GDM and covariates to Hb level. Covariates included smoking, the gestational week Hb was measured, pre-pregnancy BMI, parity, maternal age at the delivery, educational attainment and hypertensive disorders. Smoking, the gestational week Hb was measured, parity and maternal age at the delivery were included as the factors potentially affecting the Hb level of the participant. Pre-pregnancy BMI, educational attainment and hypertensive disorders reflect the metabolic status of the participant. To estimate the risk for GDM a multivariable logistic regression model was used. The first model was as unadjusted (crude) or having covariates affecting the Hb level and GDM status subsequently added to the models (models 1–3). To evaluate the models’ performance, area under receiver operating characteristic (ROC) curve was determined. To gain a deeper understanding of the effects of Hb levels on GDM risk, Models 2 and 3 were re-analysed without 1st trimester Hb levels and the gestational week Hb was measured as covariates.

Statistical analyses were calculated using IBM SPSS statistics version 27.0 (IBM Corp, Armonk, NY, USA).

## Results

### Women with GDM have higher Hb levels and higher BMI

Characteristics of the study population showed that women with GDM were older than the non-GDM controls and they had more comorbidities during pregnancy, such as pre-eclampsia and gestational hypertension (Table 1). The women with GDM also had higher 1st trimester Hb levels, pre-pregnancy BMI and systolic and diastolic bp before and after 20 weeks of pregnancy compared to non-GDM (Table 1). Newborns of women with GDM were heavier and needed more support postnatally than the non-GDM offspring (Table 1). Regarding smoking during pregnancy, the groups were comparable (Table 1). There were less women in the GDM group in the highest educational attainment category (upper-level tertiary) compared to controls but no significant differences between the other educational attainment categories (Table 1).

**Table 1:** Characteristics of the study population. The values are mean with (SD) or number (n) with (%). Total participants (n = 1828).

Parameter	GDM n = 958	non-GDM n = 870	*p*
**Maternal characteristics**			
Hb (g/L), 1st trimester	134 (9.4)	131 (9.0)	< 0.001
Pre-pregnancy BMI (kg/m^2^)	28.0 (5.98)	23.6 (4.26)	< 0.001
Maternal age (y)	31.9 (5.3)	29.4 (5.2)	< 0.001
Parity	1.19 (1.78)	0.98 (1.68)	0.011
Gravidity	1.76 (2.21)	1.38 (1.98)	< 0.001
Educational attainment	859	757	
Basic or less	56 (6.5)	35 (4.6)	0.10
Secondary	397 (46.2)	344 (45.4)	0.76
Lower-level tertiary	231 (26.9)	184 (24.3)	0.24
Upper-level tertiary	175 (18.3)	194 (25.6)	0.01
Chronic hypertension (n (%))	148 (15.4)	45 (5.2)	< 0.001
Gestational hypertension (n (%))	200 (20.9)	124 (14.3)	< 0.001
Pre-eclampsia (n (%))	57 (5.9)	23 (2.6)	< 0.001
Pharmacologically treated GDM (n (%))	170 (17.7)	0	< 0.001
Smoking during pregnancy (n (%))	163 (17.1)	125 (14.4)	0.11
Fasting glucose (mmol/L)	5.26 (0.52)	4.66 (0.29)	< 0.001
1 h glucose (OGTT) (mmol/L)	9.47 (1.85)	7.14 (1.38)	< 0.001
2 h glucose (OGTT) (mmol/L)	7.42 (1.68)	5.83 (1.07)	< 0.001
Systolic bp before 20 weeks (mmHg)	124 (12.4)	118 (10.7)	< 0.001
Diastolic bp before 20 weeks (mmHg)	76 (9.1)	72 (7.9)	< 0.001
Systolic bp after 20 weeks (mmHg)	133 (13.0)	128 (10.8)	< 0.001
Diastolic bp after 20 weeks (mmHg)	84 (9.2)	81 (8.3)	< 0.001
**Newborn characteristics**			
Gestational age (wk)	39.6 (1.40)	40.1 (1.42)	< 0.001
Birth weight (g)	3632 (503)	3564 (488)	0.004
Birth weight SD	0.21 (1.11)	− 0.12 (0.97)	< 0.001
Placental weight (g)	643 (140)	603 (121)	< 0.001
Placental weight ratio (%)	17.7 (2.87)	16.9 (2.50)	< 0.001
Apgar 1 min	8.51 (1.32)	8.60 (1.16)	0.14
Apgar 5 min	8.92 (0.85)	8.96 (0.89)	0.34
Umbilical artery pH	7.25 (0.085)	7.24 (0.083)	0.45
Umbilical vein pH	7.34 (0.077)	7.33 (0.081)	0.25
Need of intensive care	101 (10.5)	58 (6.7)	0.003
Need of any supplementary oxygen^1^ (n (%))	121 (13.0)	78 (9.2)	0.010
Need of glucose infusion (n (%))	78 (8.5)	27 (3.2)	< 0.001
Hospital stay (d)	3.83 (2.56)	3.30 (1.82)	< 0.001

^1^ Need of any supplementary oxygen or respiratory support. *GDM* gestational diabetes, *Hb* hemoglobin, *BMI* body mass index, *OGTT* oral glucose tolerance test, *bp* blood pressure, *SD* standard deviation.

### Associations of Hb levels with maternal metabolic parameters and perinatal outcome

**Figure 1:**
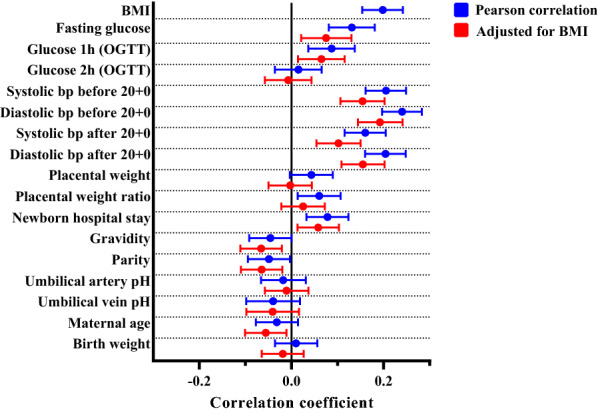
Associations of Hb levels with maternal metabolic parameters and perinatal outcome. Forest plots representing the effect sizes and their 95% confidence interval with Pearson correlation (blue) or partial correlation adjusted for BMI (red). *BMI* body mass index, *OGTT* oral glucose tolerance test, *bp* blood pressure.

We next examined the association of 1st trimester Hb levels with maternal metabolic and other parameters and perinatal outcome. There was a positive association between the Hb level and pre-pregnancy BMI (Fig. 1, Table S1). A positive association between the Hb level and fasting glucose level, 1 h glucose level in an OGTT and systolic and diastolic bp throughout pregnancy was also detected and it remained significant after adjusting for pre-pregnancy BMI (Fig. 1, Table S1). Parity and gravidity associated negatively with Hb levels independent of pre-pregnancy BMI, as did also maternal age at delivery when adjusted for pre-pregnancy BMI (Fig. 1, Table S1). The positive association between maternal Hb level and placental weight ratio was lost after adjusting for BMI while that for the newborn hospital stay stayed significant independent of pre-pregnancy BMI (Fig. 1, Table S1). No significant associations were seen with the maternal Hb levels and newborn parameters including birth weight and umbilical artery or vein pH (Fig. 1, Table S1).

### Characteristics of the participants in four Hb quartiles

The study participants were then divided to four quartiles according to their 1st trimester Hb levels with Hb quartile 1 having the lowest and Hb quartile 4 the highest levels. The women in Hb quartiles 2, 3 and 4 had higher prevalence of GDM, gestational hypertension and chronic hypertension compared to Hb quartile 1 (Table 2). Systolic and diastolic bp showed a significant dose-dependent increase throughout pregnancy in Hb quartiles 2, 3 and 4 and in Hb quartile 4 these associations retained their significance after adjusting for smoking, the gestational week Hb level was measured, pre-pregnancy BMI and chronic hypertension (Table 2). An identical dose dependent increase in pre-pregnancy BMI and fasting glucose levels was seen in Hb quartiles 2–4, with Hb quartile 4 showing the most adverse values (Table 2). When smoking and the gestational week Hb was measured were used as covariates, the pre-pregnancy BMI and fasting glucose levels remained significantly higher in Hb quartile 4 (Table 2). The 1 h glucose levels in an OGTT were the highest in the Hb quartile 4 and remained statistically significant after adjusting for smoking, the gestational week Hb was measured and pre-pregnancy BMI (Table 2). The newborn need for any supplementary oxygen or respiratory support or intravenous glucose administration and the days the newborn stayed in hospital were the highest in Hb quartile 4 (Table 2). No differences between the Hb quartiles were seen in the other newborn parameters including birth weight, gestational age, placental weight or the Apgar scores (Table 2).

**Table 2:** Characteristics of the study population in four Hb quartiles. The values are mean with (SD) or number (n) with (%). Total participants (n = 1828).

Parameter	Hb quartile 1	Hb quartile 2	Hb quartile 3	Hb quartile 4
**Maternal characteristics**				
Number of participants	466	427	460	475
Hb (g/L)	121 (4.24)	130 (1.68)*	135 (1.75)*	145 (4.13)*^φ^
GDM (n (%))	199 (42.7)	212 (49.6)	241 (52.4)	306 (64.4)*
Pharmacologically treated GDM (n (%))	39 (8.4)	35 (8.2)	38 (8.3)	58 (12.2)
Pre-pregnancy BMI (kg/m^2^)	24.2 (4.91)	25.9 (5.66)*	26.2 (5.91)*	27.4 (5.76)*^γ^
Maternal age (y)	30.8 (5.36)	30.8 (5.31)	30.4 (5.55)	30.6 (5.23)
Chronic hypertension (n (%))	26 (5.6)	50 (11.7)	43 (9.3)	74 (15.6)*
Gestational hypertension (n (%))	59 (12.7)	70 (16.4)	85 (18.5)	110 (23.2)*
Pre-eclampsia (n (%))	17 (3.6)	19 (4.4)	18 (3.9)	26 (5.5)
Parity	1.18 (1.97)	1.04 (1.76)	1.11 (1.65)	1.03 (1.57)
Gravidity	1.65 (2.38)	1.54 (2.13)	1.61 (2.05)	1.52 (1.85)
Fasting glucose (mmol/L)	4.92 (0.50)	5.04 (0.53)*	5.06 (0.51)*	5.11 (0.57)*^γ^
1 h glucose (OGTT) (mmol/L)	8.32 (2.08)	8.56 (2.02)	8.57 (2.04)	8.89 (1.96)*^φ^
2 h glucose (OGTT) (mmol/L)	6.78 (1.63)	6.87 (1.68)	6.82 (1.67)	6.86 (1.71)
Systolic bp before 20 weeks (mmHg)	118 (12.2)	121 (12.1)*	121 (10.6)*	125 (12.0)*ˤ
Diastolic bp before 20 weeks (mmHg)	71 (8.4)	74 (9.1)*	74 (8.2)*	77 (8.7)*ˤ
Systolic bp after 20 weeks (mmHg)	128 (12.5)	131 (12.9)*	130 (10.9)*	134 (12.4)*ˤ
Diastolic bp after 20 weeks (mmHg)	80 (8.8)	83 (9.2)*	82 (8.1)*	85 (9.0)*ˤ
**Newborn characteristics**				
Gestational age (wk)	39.9 (1.38)	39.9 (1.44)	39.9 (1.45)	39.7 (1.45)
Birth weight (g)	3584 (499)	3596 (513)	3632 (493)	3588 (484)
Birth weight SD	-0.0016 (1.05)	0.049 (1.08)	0.10 (1.03)	0.070 (1.08)
Placental weight (g)	618 (127)	622 (139)	632 (134)	625 (130)
Placental weight ratio (%)	17.2 (2.54)	17.3 (2.73)	17.4 (2.86)	17.5 (2.76)
Apgar 1 min	8.62 (1.17)	8.49 (1.36)	8.60 (1.22)	8.50 (1.26)
Apgar 5 min	8.95 (0.80)	8.96 (0.81)	8.96 (0.94)	8.90 (0.90)
Umbilical artery pH	7.24 (0.085)	7.24 (0.084)	7.25 (0.083)	7.24 (0.085)
Umbilical vein pH	7.34 (0.080)	7.33 (0.082)	7.34 (0.073)	7.33 (0.080)
Need of any supplementary oxygen^1^ (n (%))	45 (9.9)	46 (11.0)	41 (9.2)	67 (14.6)*
Need of glucose infusion (n (%))	17 (3.7)	31 (7.4)	23 (5.2)	34 (7.4)*
Hospital stay (d)	3.30 (1.54)	3.59 (2.41)	3.66 (2.67)	3.75 (2.23)*^γ^

*Statistical significance when compared to Hb quartile 1. ^γ^Adjusted for smoking and the gestational week hemoglobin was measured. ^φ^Adjusted for smoking, the gestational week hemoglobin was measured and pre-pregnancy BMI. ˤAdjusted for smoking, the gestational week hemoglobin was measured, pre-pregnancy BMI and chronic hypertension.

^1^Need of any supplementary oxygen or respiratory support. *Hb* hemoglobin, *GDM* gestational diabetes mellitus, *BMI* body mass index, *OGTT* oral glucose tolerance test, *bp* blood pressure.

### Association of Hb levels with GDM risk

**Figure 2:**
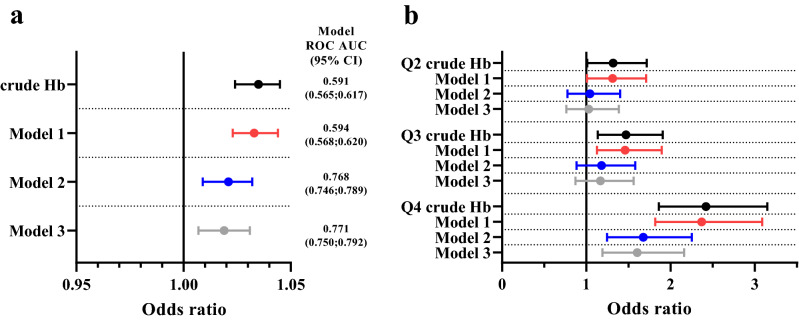
Multivariable logistic regression risk model of gestational diabetes mellitus (GDM). (**a**) Hemoglobin (Hb) as a linear variable in the logistic regression model. Crude Hb = unadjusted model. Model 1 = adjusted for smoking and the gestational week Hb was measured. Model 2 = adjusted for smoking, the gestational week Hb was measured, maternal age, parity, pre-pregnancy body mass index (BMI) and maternal educational attainment. Model 3 = adjusted for smoking, the gestational week Hb was measured, maternal age, parity, pre-pregnancy BMI, maternal educational attainment and hypertensive disorders. (**b**) Hb divided to quartiles and compared with quartile 1 with the lowest Hb level (110–126 g/L) with logistic regression. Q2 = Hb quartile 2 (Hb 127–132 g/L), Q3 = Hb quartile 3 (Hb 133–138 g/L), Q4 = Hb quartile 4 (139–155 g/l). Crude Hb = unadjusted model. Models 1–3 as in (**a**). n = 1816 in (**a**) and (**b**). Model ROC AUC = area under receiver operating characteristic curve of the models. *CI* confidence interval.

The participants with GDM showed higher Hb levels compared to non-GDM controls after adjusting for smoking, the gestational week Hb was measured, pre-pregnancy BMI, parity, maternal age, educational attainment and hypertensive disorders (Table 3). In a multivariable regression analysis Hb levels remained an independently associated parameter for GDM after adjusting for two (Model 1), six (Model 2) or seven (Model 3) of the most relevant covariates, Model 3 being adjusted for smoking, the gestational week Hb was measured, maternal age, parity, pre-pregnancy BMI, maternal educational attainment and hypertensive disorders (Fig. 2a, Table S2). When assessed in Hb quartiles, Hb quartile 4 showed increased risk for development of GDM independently from the participant’s pre-pregnancy BMI and all other covariates compared to the lowest Hb level quartile (Fig. 2b, Table S3).

**Table 3:** Linear regression model for Hb. Total participants (n = 1822).

Variable	Mean difference in Hb (g/L)	95% CI	*p* value
GDM	1.746	0.822, 2.670	< 0.001
Smoking during pregnancy	0.747	− 0.444, 1.939	0.219
Hb week	− 0.506	− 0.802, − 0.210	0.001
Pre-pregnancy BMI	0.204	0.120, 0.287	< 0.001
Parity	− 0.027	− 0.429, 0.375	0.895
Maternal age	− 0.100	− 0.189, − 0.012	0.026
Educational attainment	− 0.197	− 0.563, 0.169	0.292
Hypertensive disorders^1^	2.107	1.153, 3.061	< 0.001

^1^Hypertensive disorders include chronic hypertension, gestational hypertension and pre-eclampsia. *GDM* gestational diabetes, *Hb week* the gestational week hemoglobin was measured, *BMI* body mass index.

We then evaluated the quality of the Models by calculating the specificity, sensitivity, F_1_-score and area under the ROC curve for each developed model. Using Hb as a linear variable, crude Hb had 70.0% sensitivity and 42.0% specificity (ROC AUC 0.591, 95% CI [0.565; 0.617]) on prediction of GDM while Model 2 had the highest sensitivity (70.7%) and specificity (70.7%) (ROC AUC 0.768, 95% CI [0.746; 0.789]) (Fig. 2a, Table S4). When Hb levels were assessed in quartiles, crude Hb had sensitivity of 57.1% and specificity of 55.4% (ROC AUC 0.587, 95% CI [0.561; 0.613]), Model 3 having the highest performance with sensitivity of 70.5% and specificity of 70.0% (ROC AUC 0.772, 95% CI [0.750; 0.793] (Table S4). When Hb levels were removed from the Models 2 and 3 as a linear variable in multivariable logistic regression it resulted in a decline in ROC AUCs (0.768 vs. 0.763 in Model 2 and 0.771 vs. 0.768 in Model 3) (Table S4). Therefore, addition of the 1st trimester Hb levels to Models slightly improved their performance in predicting the GDM risk, being in line with the role of Hb as a tissue oxygenation marker and likely contributing to maternal metabolism.

### Characteristics of the participants with GDM in four Hb quartiles

When participants with GDM were divided to four quartiles based on their 1st trimester Hb level, with Hb quartile 1 having the lowest and Hb quartile 4 the highest levels, no statistical significance was detected in the prevalence of pharmacologically treated GDM between the Hb quartiles (Table 4). Although there was a tendency for gestational hypertension to increase with the Hb level, similarly to the full study population, only the prevalence of chronic hypertension reached significance between the quartiles being higher in Hb quartile 4 (Tables 2, 4). Pre-pregnancy BMI, fasting glucose level and bp parameters differed significantly between the Hb quartiles among the participants with GDM, participants in Hb quartile 4 having the most adverse values, and a dose-dependent increase being detected for pre-pregnancy BMI and diastolic bp throughout pregnancy (Table 4). After adjusting for smoking, the gestational week Hb was measured, pre-pregnancy BMI and chronic hypertension, diastolic bp before and after mid-pregnancy was higher in Hb quartile 4, as was systolic bp before 20 weeks (Table 4). No differences between the Hb quartiles were seen with the newborn parameters among participants with GDM (Table 4).

**Table 4:** Characteristics of the participants with GDM in different Hb quartiles. The values are mean with (SD) or number (n) with (%). Total participants (n = 958).

Variable	Hb quartile 1	Hb quartile 2	Hb quartile 3	Hb quartile 4
**Maternal characteristics**				
Number of participants	254	237	213	254
Hb (g/L)	122 (4.75)	132 (1.63)*	137 (1.63)*	146 (3.65)*^φ^
Pre-pregnancy BMI (kg/m^2^)	26.7 (6.00)	28.3 (5.94)*	28.2 (6.04)*	28.9 (5.76)*^γ^
Maternal age (y)	32.3 (5.25)	31.8 (5.26)	31.7 (5.47)	31.6 (5.15)
Pharmacologically treated GDM (n (%)	44 (19.6)	44 (16.4)	30 (14.1)	53 (20.8)
Chronic hypertension (n (%))	22 (9.8)	46 (17.2)	25 (11.7)	55 (21.6)*
Gestational hypertension (n (%))	36 (16.1)	53 (19.8)	49 (23.0)	62 (24.3)
Pre-eclampsia (n (%))	12 (5.4)	16 (6.0)	12 (5.6)	18 (7.1)
Parity	1.30 (2.24)	1.14 (1.58)	1.11 (1.48)	1.19 (1.68)
Gravidity	1.87 (2.68)	1.72 (2.11)	1.71 (2.02)	1.74 (1.92)
Fasting glucose (mmol/L)	5.18 (0.50)	5.28 (0.51)	5.27 (0.47)	5.31 (0.57)*^γ^
1 h glucose (OGTT) (mmol/L)	9.58 (1.83)	9.17 (1.97)	9.54 (1.82)	9.57 (1.77)
2 h glucose (OGTT) (mmol/L)	7.54 (1.55)	7.38 (1.68)	7.45 (1.76)	7.30 (1.75)
Systolic bp before 20 weeks (mmHg)	121 (13.2)	124 (12.4)	123 (10.6)	127 (12.2)*ˤ
Diastolic bp before 20 weeks (mmHg)	73 (9.0)	77 (9.2)*	77 (8.2)*	79 (9.1)*ˤ
Systolic bp after 20 weeks (mmHg)	131 (14.2)	133 (12.2)	133 (12.0)	136 (13.0)*^φ^
Diastolic bp after 20 weeks (mmHg)	82 (9.7)	85 (8.8)*	84 (8.3)*	86 (9.2)*ˤ
**Newborn characteristics**				
Gestational age (wk)	39.6 (1.48)	39.7 (1.38)	39.7 (1.31)	39.5 (1.40)
Birth weight (g)	3625 (536)	3674 (499)	3604 (499)	3624 (474)
Birth weight SD	0.192 (1.15)	0.299 (1.08)	0.0995 (1.09)	0.241 (1.13)
Placental weight (g)	636 (139)	657 (138)	631 (146)	647 (135)
Placental weight ratio (%)	17.5 (2.68)	17.8 (2.93)	17.6 (3.12)	17.9 (2.76)
Apgar 1 min	8.42 (1.54)	8.47 (1.43)	8.65 (0.96)	8.53 (1.24)
Apgar 5 min	8.85 (0.96)	8.89 (0.95)	9.03 (0.65)	8.92 (0.79)
Umbilical artery pH	7.24 (0.092)	7.24 (0.087)	7.26 (0.079)	7.25 (0.082)
Umbilical vein pH	7.34 (0.085)	7.33 (0.074)	7.35 (0.066)	7.34 (0.079)
Need of any supplementary oxygen^1^ (n (%))	28 (12.8)	33 (12.5)	20 (9.8)	40 (16.3)
Need of glucose infusion (n (%))	15 (6.9)	26 (10.0)	15 (7.4)	22 (9.0)
Hospital stay (d)	3.58 (1.77)	4.05 (3.09)	3.92 (3.12)	3.79 (2.10)

*Statistical significance when compared to Hb quartile 1. ^γ^Adjusted for smoking and the gestational week hemoglobin was measured. ^φ^Adjusted for smoking, the gestational week hemoglobin was measured and pre-pregnancy BMI. ˤAdjusted for smoking, the gestational week hemoglobin was measured, pre-pregnancy BMI and chronic hypertension. ^1^Need of any supplementary oxygen or respiratory support. *GDM* gestational diabetes mellitus, *Hb* hemoglobin, *BMI* body mass index, *OGTT* oral glucose tolerance test, *bp* blood pressure.

## Discussion

In the current case–control study we showed that within the Finnish reference values, a higher 1st trimester Hb level is an independent risk factor for development of GDM. Especially, the highest Hb quartile (Hb quartile 4, 141–155 g/L) showed an increased risk for GDM (OR 1.604 95% CI [1.192; 2.160]) which was independent of maternal pre-pregnancy BMI levels. These results are in line and in scale with most of the previous studies including two large studies (n > 20,000 and n > 360,000) with East Asian subjects^15–19,25,26^, while some small studies with mixed ancestry or Iranian subjects failed to show an increased risk for GDM with higher Hb levels^27–29^ (Table 5). On the other hand, there are a few studies that have reported a decreased risk for GDM in anemic pregnancies^30,31^. Nutritional status has been considered the most relevant factor underlaying the relation between Hb levels and GDM^15–19,30,31^, poor nutritional status being a factor behind anemia and better nutritional status leading to obesity and metabolic dysfunction. Also, iron overload and increased oxidative stress leading to inhibition of insulin internalization and function can result to hyperinsulinemia and insulin resistance with high Hb levels^32^.

**Table 5:** Summary of earlier studies presenting data on the association of 1st trimester Hb levels with the risk for GDM. Included were studies reporting odds ratio (OR) with 95% confidence intervals (CI).

Study	Ancestry	n	Hb groups	Crude OR (95% CI)	Adjusted OR (95% CI)	Covariates
Lao et al. 2002^16^	Chinese	762 (GDM, n = 94)	> 130 g/L vs. < 130 g/L	1.87 (1.18; 2.96)	1.73 (1.08; 2.78)	Pre-pregnancy BMI, maternal age, parity
Chen et al. 2006^27^	US Hispanic, African American, Caucasian, Asian	1456 (GDM, n = 45)	> 130 g/L vs. < 130 g/L		0.81 (0.36; 1.81)	Maternal age, ethnicity, parity, family history of diabetes
Wang et al. 2018^19^	Chinese	21,577 (GDM, n = 4337)	75–182 g/L	1.019 (1.016; 1.023)	1.015 (1.1011; 1.018)	Maternal age, pre-pregnancy BMI, gravidity, parity, educational level, the gestational week hemoglobin was measured, family history of diabetes
Behboudi-Gandevani et al. 2013^28^	Iranian	1033 (GDM, n = 72)	Assessed as linear, mean 115 g/L		1 (0.8; 1.3)	Maternal age, pre-pregnancy BMI, educational level, parity, passive smoking, history of gestational diabetes and/or family diabetes, serum zinc/iron levels and deficient intakes
Soheilykhah et al. 2017^29^	Iranian	1358 (GDM, n = 300)	> 135 g/L vs. < 135 g/L	1.19 (0.88; 1.6)		
Kim et al. 2021^26^	Korean	366,122 (GDM, n = 14,799)	> 130 g/L vs. 110–130 g/L (pre-pregnancy)		1.185 (1.145; 1.226)	Maternal age, parity, smoking, pre-pregnancy BMI, hypertension, fasting glucose, high AST, high ALT, high total cholesterol
Li et al. 2021^25^	Chinese	1360 (GDM, n = 343)	> 150 g/L vs. 115–150 g/L	2.09 (1.49; 2.92)	1.92 (1.27; 2.88)	Maternal age, pre-pregnancy BMI, parity, HbA1c, fasting glucose, ALT and AST concentrations

*Hb* hemoglobin, *GDM* gestational diabetes mellitus, *BMI* body mass index, *ALT* alanine aminotransferase, *AST* aspartate aminotransferase.

Our group has recently shown that within the normal variation of Hb levels, lower Hb levels could be used as a surrogate measure for hypoxia^13^. The lower Hb levels associated with low-grade activation of the hypoxia-inducible factor (HIF) mediated transcriptional response, the HIF prolyl 4-hydroxylases (HIF-P4Hs) acting as the oxygen sensing component in this pathway. The HIF-mediated reprogramming of energy metabolism, leading to promotion of insulin-independent glucose intake and upregulation of the non-oxygen demanding glycolytic metabolism and downregulation of oxidative phosphorylation, has been shown to mediate protection against obesity and metabolic syndrome resulting in reduced body weight and adiposity, lower serum cholesterol levels, less white adipose tissue inflammation and improved glucose tolerance and insulin sensitivity in human and mice, also during gestation^13,33,34^. Combining the results of this study, we offer HIF activation as a potential mechanism behind the protective effect of the lower-end normal Hb levels and decreased risk for GDM.

During pregnancy hemodilution is a physiological phenomenon to improve blood flow to the uterus and developing fetus. Hemodilution starts already during the 1st trimester, Hb reaching its lowest levels around gestational week 20 after which the Hb levels starts to rise reaching the pre-pregnancy level around gestational week 30 if the nutritional status allows^35^. The maternal Hb levels have a u-shaped association to adverse pregnancy disorders and outcomes^36^. The Hb levels here were measured during the first visit (average gestational week 9.26) to a maternity clinic with point-of-care testing. Timing of the first visit hence affects the Hb level measured which was used as a covariate in many analyses. In Finland, iron supplementation is provided to all mothers with Hb levels < 110 g/L before the 12th gestational week or after 28th gestational week if the Hb levels decline to < 105 g/L^37^. The participants’ iron status in this study was unknown but the participants with pre-existing iron deficiency were excluded from the analyses based on the set lower-end Hb limit (110 g/L).

A positive association between Hb levels and BMI that is independent of sex or age has earlier been reported in Finnish cohorts^13,38^ and was replicated here. The association was seen also in the Hb quartile comparisons, where the pre-pregnancy BMI increased Hb dose-dependently. Pre-pregnancy BMI is a strong predictor for the development of insulin resistance, DM and GDM. Since the Hb levels are an independent risk factor for GDM and undeniably associate with BMI, the underlaying effect of Hb to the development of GDM and maternal health might be even greater.

The association of higher Hb levels with gestational hypertension is well-established^36,39^. In addition our group has shown in three separate Finnish cohorts the association between Hb levels and bp parameters and other cardiovascular risk factors^13,38^. In this study we showed the same association in pregnant women. Bp values rose dose-dependently regarding Hb levels. Focusing only to participants with GDM the same effect was seen. Here, the association of bp levels with the Hb levels was the most sustained association after adjusting for the GDM covariates. Within the participants with GDM the association of Hb levels with systolic bp after mid-pregnancy lost its significance after adjusting for covariates. This could reflect the high prevalence of gestational hypertension and medications started during pregnancy which were not included as covariates. It was shown that participants with hypertensive disorders during pregnancy had higher Hb values compared to non-hypertensives, mean difference being 2.107 g/L (95% CI [1.153; 3.061]).

Concentrating only to participants with GDM there was a significant difference between the Hb quartiles in fasting glucose values which were mainly explained by the higher BMI. Interestingly, there was no significant difference between the Hb quartiles and the severity of GDM based on the proportion of pharmacologically treated participants with GDM.

Surprisingly, there was no significant association with maternal Hb levels and birth weight. Hypoxia is a known risk factor for intrauterine growth retardation^40^ and both anemia and high Hb levels associate with increased risk for small-for-gestational age^36^. In line with the previous results^15^ there was no association with the higher 1st trimester Hb levels and adverse neonatal outcomes. However, newborns of mothers in the highest Hb quartile had a higher need for any supplementary oxygen or respiratory support and glucose administration, which reflects the higher number of participants with GDM in that group. Also, the newborn’s hospital stay was longer in the highest Hb quartile reflecting the higher prevalence of GDM and the increased need for neonatal intensive care. Although gestational age is a marked explanator behind this difference there were no significant differences in gestational age between the Hb quartiles. Participants with GDM had a shorter gestation due to inductions in earlier stage of gestation and likely higher proportion of Cesarean sections affecting the longer hospital stay.

In a case–control study, like the current one, the study population gives limitations to applicability of the results. The participants with GDM were significantly older (by average of 2.5 years), were less-often educated to upper-level tertiary and had more adverse metabolic parameters to start with. The highest risk factor for development of GDM is a previous GDM. This study population included 870 primigravida for whom such information is not available. Polycystic ovary syndrome has previously been reported as a BMI-dependent risk factor for GDM in this study population^21^.

Risk for GDM is affected by ethnicity^22^ and to our knowledge this is the first study assessing the connection of 1st trimester Hb levels and risk for development of GDM in a population of European ancestry (Table 5). The size of the study population (n = 1828) gave an opportunity to widely assess different parameters from both mothers and newborns and combine these data in a unique way. The case–control setting offered an opportunity to also assess parameters concentrating to GDM group. In our study there were 958 participants with GDM as in previous studies the numbers have been significantly less (Lao et al*.* n = 94, Tarim et al*.* n = 20, Phaloprakarn et al*.* n = 65), only the large Chinese study exceeded the number of mothers with GDM in our study with n = 4337^16–19^. In here we assessed the Hb level both as a continuous variable and in four quartiles within the normal variation of the Finnish reference values providing information from Hb levels which did not include anemic or erythrocytic individuals.

In conclusion, higher Hb levels in the 1st trimester associated with an adverse metabolic profile leading to increased risk for GDM. Normal variation of Hb levels did not have a marked association with the perinatal health of the newborn. In reverse, lower-end normal Hb levels could protect from development of GDM likely by inducing slight tissue hypoxia and HIF activation. The latter could also be established by HIF-P4H inhibitors which are a novel group of pharmaceuticals, the first compounds Roxadustat (Evrenzo) and Daprodustat (Duvaq), recently approved in Asia and Europe for treatment of renal anemia. The clinical studies have reported in addition to correction of anemia beneficial effects to serum cholesterol levels and lipid profile, and also indications of improved glucose tolerance and decreased bp values^41–44^. GDM is a strong predictor for metabolic problems later in life and targeting the HIF-P4H inhibitor treatment to this high-risk group after pregnancy could provide a novel treatment option to improve the metabolic health of these women and decrease the burden for public health.

## Supplementary Information

Supplementary Information.

## Abbreviations

ANOVA: Analysis of variance
bp: Blood pressure
BMI: Body mass index
DM: Type 2 diabetes mellitus
FINNGEDI: The Finnish Gestational Diabetes study
GLM: General linear model
GDM: Gestational diabetes mellitus
HIF: Hypoxia-inducible factor
HIF-P4H: HIF prolyl 4-hydroxylase
MBR: The Finnish Medical Birth Register
OGTT: Oral glucose tolerance test
OR: Odds ratio
SD: Standard deviation
THL: The Finnish Institute for Health and Welfare

## References

[1] Damm P (2016). Gestational diabetes mellitus and long-term consequences for mother and offspring: A view from Denmark. Diabetologia.

[2] McIntyre HD (2019). Gestational diabetes mellitus. Nat. Rev. Dis. Primers.

[3] Szmuilowicz ED, Josefson JL, Metzger BE (2019). Gestational diabetes mellitus. Endocrinol. Metab. Clin. North Am..

[4] Zhu Y, Zhang C (2016). Prevalence of gestational diabetes and risk of progression to type 2 diabetes: A global perspective. Curr. Diabetes Rep..

[5] Tiitinen A (2021). Gestational Diabetes.

[6] The Finnish Institute for Health (THL). *Perinatal statistics—parturients, deliveries and newborns 2019*. *Official Statistics of Finland, Perinaltal statistics*, Vol. 48/2020. https://www.julkari.fi/handle/10024/140702 (2020).

[7] Vääräsmäki M (2016). Is it worth treating gestational diabetes: If so, when and how?. Diabetologia.

[8] Zhang C, Rawal S, Chong YS (2016). Risk factors for gestational diabetes: Is prevention possible?. Diabetologia.

[9] van der Harst P (2012). Seventy-five genetic loci influencing the human red blood cell. Nature.

[10] Patel KV (2008). Variability and heritability of hemoglobin concentration: An opportunity to improve understanding of anemia in older adults. Haematologica.

[11] World Health Organization. *Haemoglobin concentrations for the diagnosis of anaemia and assessment of severity*. https://apps.who.int/iris/handle/10665/85839 (2011).

[12] Atsma F (2012). Hemoglobin level is positively associated with blood pressure in a large cohort of healthy individuals. Hypertension.

[13] Auvinen J (2021). Systematic evaluation of the association between hemoglobin levels and metabolic profile implicates beneficial effects of hypoxia. Sci. Adv..

[14] Hashimoto Y (2015). Hemoglobin concentration and incident metabolic syndrome: A population-based large-scale cohort study. Endocrine.

[15] Abeysena C, Jayawardana P, de A Seneviratne R (2010). Maternal haemoglobin level at booking visit and its effect on adverse pregnancy outcome. Aust. N. Z. J. Obstet. Gynaecol..

[16] Lao TT, Chan LY, Tam KF, Ho LF (2002). Maternal hemoglobin and risk of gestational diabetes mellitus in Chinese women. Obstet. Gynecol..

[17] Phaloprakarn C, Tangjitgamol S (2008). Impact of high maternal hemoglobin at first antenatal visit on pregnancy outcomes: A cohort study. J. Perinat. Med..

[18] Tarim E, Kilicdag E, Bagis T, Ergin T (2004). High maternal hemoglobin and ferritin values as risk factors for gestational diabetes. Int. J. Gynaecol. Obstet..

[19] Wang C (2018). Hemoglobin levels during the first trimester of pregnancy are associated with the risk of gestational diabetes mellitus, pre-eclampsia and preterm birth in Chinese women: A retrospective study. BMC Pregnancy Childbirth.

[20] Keikkala E (2020). Cohort profile: The Finnish Gestational Diabetes (FinnGeDi) study. Int. J. Epidemiol..

[21] Mustaniemi S (2018). Polycystic ovary syndrome and risk factors for gestational diabetes. Endocr. Connect..

[22] Working Group Established by the Finnish Medical Society Duodecim, the M. A. B. of the F. D. A. and the F. G. Association (2008). Current Care Guideline. Gestational Diabetes.

[23] Kairisto V (2003). New Finnish reference limits for basic blood count. Suom. Laakaril..

[24] Sankilampi U, Hannila ML, Saari A, Gissler M, Dunkel L (2013). New population-based references for birth weight, length, and head circumference in singletons and twins from 23 to 43 gestation weeks. Ann. Med..

[25] Li M, Hu M, Yue Z, Zhang Y, Yang H (2021). The interactive effects of non-alcoholic fatty liver disease and hemoglobin concentration in the first trimester on the development of gestational diabetes mellitus. PLoS One.

[26] Kim HY (2021). Prepregnancy hemoglobin levels and gestational diabetes mellitus in pregnancy. Diabetes Res. Clin. Pract..

[27] Chen X, Scholl TO, Stein TP (2006). Association of elevated serum ferritin levels and the risk of gestational diabetes mellitus in pregnant women. The Camden study. Diabetes Care.

[28] Behboudi-Gandevani S (2013). The relationship between maternal serum iron and zinc levels and their nutritional intakes in early pregnancy with gestational diabetes. Biol. Trace Elem. Res..

[29] Soheilykhah S, Mojibian M, Moghadam MJ (2017). Serum ferritin concentration in early pregnancy and risk of subsequent development of gestational diabetes: A prospective study. Int. J. Reprod. Biomed..

[30] Lao TT, Ho LF (2004). Impact of iron deficiency anemia on prevalence of gestational diabetes mellitus. Diabetes Care.

[31] Jung J (2019). Effects of hemoglobin levels during pregnancy on adverse maternal and infant outcomes: A systematic review and meta-analysis. Ann. N. Y. Acad. Sci..

[32] Fernández-Real JM, López-Bermejo A, Ricart W (2002). Cross-talk between iron metabolism and diabetes. Diabetes.

[33] Määttä J (2018). Hypoxia causes reductions in birth weight by altering maternal glucose and lipid metabolism. Sci. Rep..

[34] Rahtu-Korpela L (2014). HIF prolyl 4-hydroxylase-2 inhibition improves glucose and lipid metabolism and protects against obesity and metabolic dysfunction. Diabetes.

[35] Pritchard JA (1965). Changes in the blood volume during pregnancy and delivery. Anesthesiology.

[36] Murphy JF, O’Riordan J, Newcombe RG, Coles EC, Pearson JF (1986). Relation of haemoglobin levels in first and second trimesters to outcome of pregnancy. Lancet (London, England).

[37] Klemetti R, Hakulinen-Viitanen T (2013). Äitiysneuvolaopas—Suosituksia äitiysneuvolatoimintaan (Recomendations for Maternal Health Center).

[38] Tapio J, Vähänikkilä H, Kesäniemi YA, Ukkola O, Koivunen P (2021). Higher hemoglobin levels are an independent risk factor for adverse metabolism and higher mortality in a 20-year follow-up. Sci. Rep..

[39] Huisman A, Aarnoudse JG (1986). Increased 2nd trimester hemoglobin concentration in pregnancies later complicated by hypertension and growth retardation. Early evidence of a reduced plasma volume. Acta Obst. Gynecol. Scand..

[40] Jensen GM, Moore LG (1997). The effect of high altitude and other risk factors on birthweight: Independent or interactive effects?. Am. J. Public Health.

[41] Flamme I (2014). Mimicking hypoxia to treat anemia: HIF-stabilizer BAY 85–3934 (Molidustat) stimulates erythropoietin production without hypertensive effects. PLoS ONE.

[42] Maxwell PH, Eckardt K-U (2016). HIF prolyl hydroxylase inhibitors for the treatment of renal anaemia and beyond. Nat. Rev. Nephrol..

[43] Olson E (2014). Short-term treatment with a novel HIF-prolyl hydroxylase inhibitor (GSK1278863) failed to improve measures of performance in subjects with claudication-limited peripheral artery disease. Vasc. Med..

[44] Provenzano R (2016). Oral hypoxia-inducible factor prolyl hydroxylase inhibitor Roxadustat (FG-4592) for the treatment of anemia in patients with CKD. Clin. J. Am. Soc. Nephrol..

