# Supplementary material for: Higher hemoglobin levels are an independent risk factor for gestational diabetes
Source: Sci Rep. 2022 Jan 31;12:1686. doi: 10.1038/s41598-022-05801-y (PMC8803843; doi:10.1038/s41598-022-05801-y)
Supplement: Supplementary file 1 — Supplementary Information. [file 41598_2022_5801_MOESM1_ESM.pdf]

SUPPLEMENTAL MATERIAL

Higher hemoglobin levels are an independent risk factor for gestational diabetes

Niina Sissala[1], MD; Sanna Mustaniemi[2,3], MD; Eero Kajantie[2,3,4,5], MD, PhD; Marja Vääräsmäki[2,3], MD, PhD; Peppi Koivunen[1,*], MD, PhD

[1]Biocenter Oulu and Faculty of Biochemistry and Molecular Medicine, Oulu Center for Cell-Matrix Research, University of Oulu, 90014 Oulu, Finland. 90014 Oulu, Finland.

[2]PEDEGO Research Unit, MRC Oulu, Oulu University Hospital and University of Oulu; Oulu, Finland

[3]Population Health Unit, Finnish Institute for Health and Welfare, Helsinki and Oulu, Finland

[4]Department for Clinical and Molecular Medicine, Norwegian University of Science and Technology, Trondheim, Norway

[5]Children's Hospital, Helsinki University Hospital and University of Helsinki, Helsinki, Finland

*To whom correspondence should be addressed: Peppi Koivunen, MD, PhD, Faculty of Biochemistry and Molecular Medicine, University of Oulu, P.O. Box 5400, FIN-90014 University of Oulu, Oulu, Finland, Email: peppi.koivunen@oulu.fi,Tel. +358294485822.

Key words: Gestational diabetes, glucose tolerance**,** hemoglobin, hypertension, hypoxia

Running title: Hemoglobin and gestational diabetes

Supplemental tables

Table S1 Correlation coefficients and associations of Hb levels with maternal metabolic parameters and perinatal outcome. Number of participants (N) in the statistical analyses, correlation coefficients (*r*) for each parameter and their 95% confidence intervals and *p* values.

		Pearson correlation				Partial correlation			
**Parameter**	**N**	***r***	**CIL**	**CIU**	***p***	***r***	**CIL**	**CIU**	***p***
**Fasting glucose (OGTT) (mmol/L)**	1498	0.131	0.081	0.180	<0.001	0.076	0.021	0.13	0.007
**1h glucose (OGTT) (mmol/L)**	1490	0.087	0.036	0.137	0.001	0.065	0.014	0.115	0.012
**2h glucose (OGTT) (mmol/L)**	1494	0.015	-0.036	0.065	0.575	-0.007	-0.058	0.043	0.779
**Pre-pregnancy BMI (kg/m^2)**	1827	0.198	0.154	0.242	<0.001				
**Systolic bp before 20 weeks (mmHg)**	1825	0.205	0.161	0.249	<0.001	0.154	0.106	0.202	<0.001
**Diastolic bp before 20 weeks (mmHg)**	1825	0.240	0.196	0.283	<0.001	0.192	0.144	0.241	<0.001
**Systolic bp after 20 weeks (mmHg)**	1824	0.160	0.115	0.204	<0.001	0.102	0.054	0.15	<0.001
**Diastolic bp after 20 weeks (mmHg)**	1824	0.204	0.160	0.248	<0.001	0.155	0.108	0.202	<0.001
**Placental weight (g)**	1766	0.043	-0.004	0.090	0.07	-0.003	-0.05	0.044	0.888
**Placental weight ratio (%)**	1766	0.060	0.013	0.106	0.011	0.025	-0.022	0.072	0.294
**Newborn hospital stay (d)**	1823	0.078	0.032	0.123	0.001	0.058	0.013	0.103	0.012
**Gravidity**	1828	-0.046	-0.092	0.000	0.049	-0.066	-0.111	-0.021	0.004
**Parity**	1828	-0.049	-0.095	-0.003	0.034	-0.065	-0.11	-0.02	0.005
**Umbilical artery pH**	1623	-0.018	-0.066	0.031	0.479	-0.011	-0.058	0.037	0.655
**Umbilical vein pH**	1127	-0.040	-0.099	0.018	0.175	-0.041	-0.098	0.016	0.158
**Maternal age (y)**	1828	-0.032	-0.077	0.014	0.175	-0.056	-0.101	-0.011	0.015
**Birth weight (g)**	1828	0.010	-0.036	0.056	0.667	-0.019	-0.065	0.026	0.402

Hb=hemoglobin, CIL= lower limit confidence interval, CIU=upper limit confidence interval, OGTT=oral glucose tolerance test, BMI=body mass index, bp=blood pressure.

Table S2 Multivariable logistic regression risk model of gestational diabetes mellitus with Hb as a linear variable. Odds ratio (OR) with 95% confidence intervals. Total participants (n=1822).

	Crude			Model 1			Model 2			Model 3		
**Variable**	**OR**	**CIL**	**CIU**	**OR**	**CIL**	**CIU**	**OR**	**CIL**	**CIU**	**OR**	**CIL**	**CIU**
**Hb**	1.035	1.024	1.045	1.033	1.023	1.044	1.021	1.009	1.032	1.019	1.007	1.031
**Smoking during pregnancy**				1.166	0.902	1.508	1.265	0.929	1.722	1.251	0.918	1.706
**Hb week**				0.953	0.892	1.018	0.933	0.865	1.006	0.926	0.858	1.000
**Pre-pregnancy BMI**							1.173	1.146	1.201	1.163	1.135	1.191
**Maternal age**							1.118	1.091	1.144	1.115	1.089	1.142
**Hypertensive disorder[1]**										1.472	1.158	1.872
**Educational attainment[2]**												
**Not known**							0.736	0.499	1.086	0.739	0.501	1.089
**Basic or less**							1.851	1.038	3.302	1.839	1.027	3.291
**Secondary**							1.213	0.896	1.643	1.191	0.878	1.615
**Lower-level tertiary**							1.151	0.843	1.571	1.131	0.828	1.546
**Parity[3]**												
**1**							0.912	0.711	1.171	0.965	0.749	1.243
**2**							0.762	0.537	1.083	0.818	0.574	1.165
**3 or more**							0.795	0.543	1.163	0.827	0.564	1.212

Hb=Hemoglobin, CIL= lower limit confidence interval, CIU=upper limit confidence interval, Hb week=the gestational week Hb was measured, BMI=body mass index, [1]Hypertensive disorders include chronic hypertension, gestational hypertension and pre-eclampsia. [2]Educational attainment compared to upper-level tertiary. [3]Parity compared to primipara.

Table S3 Multivariable logistic regression risk model of gestational diabetes mellitus with Hb divided to quartiles. Odds ratio (OR) with 95% confidence intervals. Total participants (n=1822).

	Crude			Model 1			Model 2			Model 3		
**Variable**	**OR**	**CIL**	**CIU**	**OR**	**CIL**	**CIU**	**OR**	**CIL**	**CIU**	**OR**	**CIL**	**CIU**
**Hb**[1]												
**Q2**	1.318	1.012	1.716	1.312	1.006	1.710	1.042	0.775	1.401	1.028	0.764	1.385
**Q3**	1.471	1.135	1.906	1.460	1.125	1.894	1.181	0.883	1.580	1.167	0.872	1.562
**Q4**	2.420	1.861	3.148	2.368	1.817	3.085	1.675	1.247	2.251	1.604	1.192	2.160
**Smoking during pregnancy**				1.187	0.917	1.535	1.275	0.936	1.736	1.261	0.925	1.719
**Hb week**				0.953	0.893	1.018	0.934	0.866	1.007	0.927	0.859	1.000
**Pre-pregnancy BMI**							1.173	1.146	1.201	1.163	1.135	1.191
**Maternal age**							1.117	1.091	1.144	1.115	1.089	1.142
**Hypertensive disorder**[2]										1.470	1.156	1.869
**Educational attainment**[3]												
**Not known**							0.736	0.499	1.086	0.738	0.500	1.088
**Basic or less**							1.870	1.048	3.336	1.853	1.035	3.318
**Secondary**							1.214	0.896	1.644	1.192	0.879	1.616
**Lower-level tertiary**							1.148	0.841	1.568	1.128	0.825	1.543
**Parity**[4]												
**1**							0.914	0.711	1.173	0.966	0.750	1.244
**2**							0.761	0.535	1.081	0.816	0.572	1.163
**3 or more**							0.789	0.540	1.154	0.821	0.560	1.204

Hb=Hemoglobin, CIL= lower limit confidence interval, CIU=upper limit confidence interval, Hb week=the gestational week Hb was measured, BMI=body mass index.[1] Hb compared to quartile 1 with the lowest Hb level. [2] Hypertensive disorders include chronic hypertension, gestational hypertension and pre-eclampsia. [3]Educational attainment compared to upper-level tertiary. [4]Parity compared to primipara

Table S4 Metrics for evaluating the performance of multivariable logistic regression risk models of gestational diabetes mellitus with Hb as a linear variable and divided to quartiles. Area under the receiver operating characteristic curve (AUC) with 95% confidence intervals. Total participants (n=1822).

**Logistic regression**	**Hb as a linear variable**						**Hb in quartiles**					
	**Sensitivity (%)**	**Specificity (%)**	**F_1-score (%)**	**AUC**	**CIL**	**CIU**	**Sensitivity (%)**	**Specificity (%)**	**F_1-score (%)**	**AUC**	**CIL**	**CIU**
**Crude**	70.0	42.0	62.9	0.591	0.565	0.617	57.1	55.4	57.8	0.587	0.561	0.613
**Model 1**	68.0	45.8	62.6	0.594	0.568	0.620	62.8	51.1	60.6	0.595	0.569	0.621
**Model 2**	70.7	70.7	71.6	0.768	0.746	0.789	70.4	70.4	71.3	0.768	0.747	0.790
**Without Hb**	69.0	70.2	70.4	0.763	0.742	0.785	69.0	70.2	70.4	0.763	0.742	0.785
**Model 3**	69.7	70.2	70.8	0.771	0.750	0.792	70.5	70.0	71.3	0.772	0.750	0.793
**Without Hb**	69.6	70.4	70.8	0.768	0.746	0.789	69.6	70.4	70.8	0.768	0.746	0.789

Hb=Hemoglobin, CIL= lower limit confidence interval, CIU=upper limit confidence interval.

.

Supplemental figures

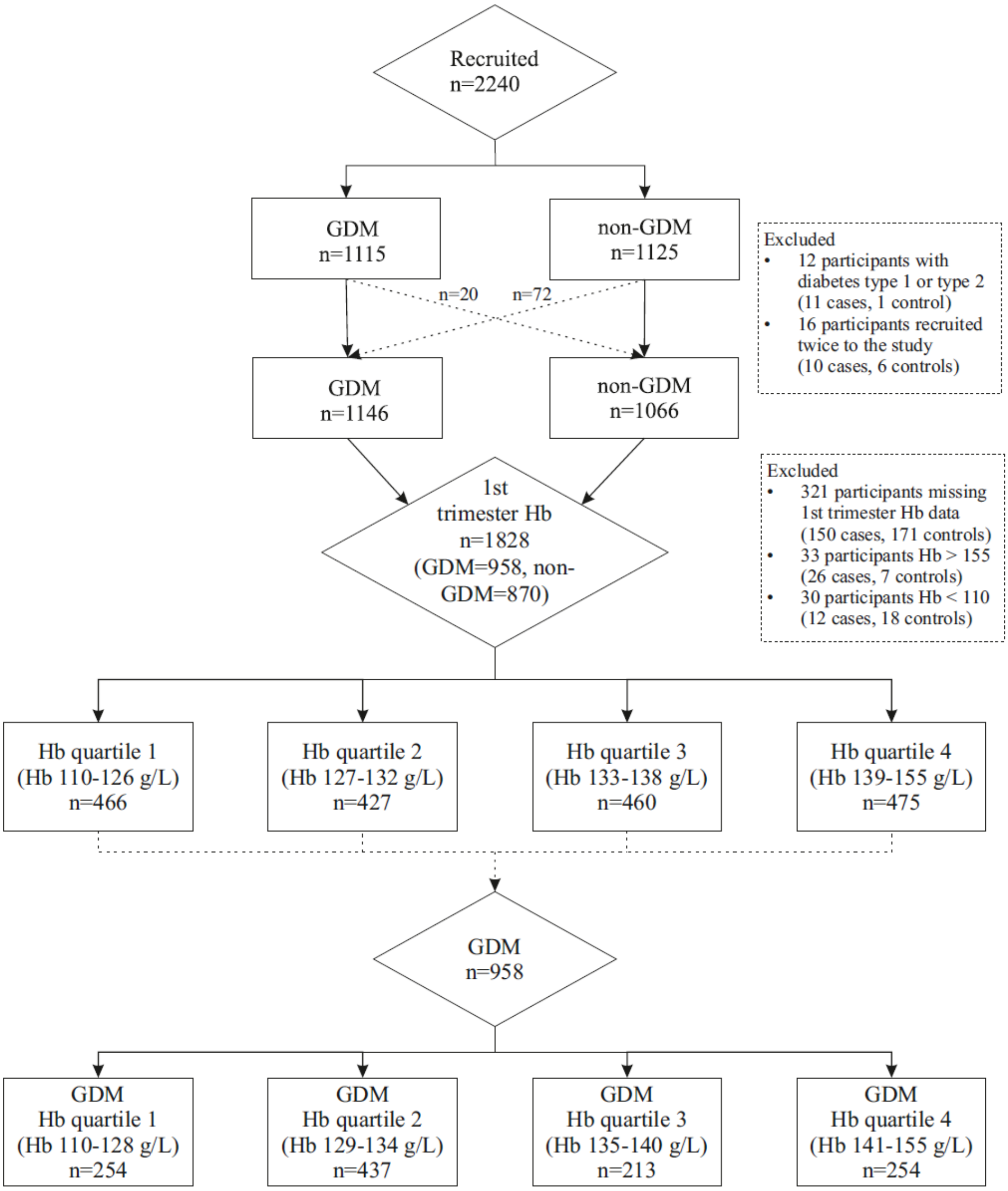

Figure S1. Flow-chart representing the study population. GDM=gestational diabetes mellitus, Hb=hemoglobin. 20 women were recruited as GDM cases but were confirmed to have normal glucose tolerance during pregnancy by medical record review. 72 women recruited as a non-GDM controls fulfilled the criteria for GDM by medical record review.
